# Structural Complexity as a Directional Signature of System Evolution: Beyond Entropy

**DOI:** 10.3390/e27090925

**Published:** 2025-09-03

**Authors:** Donglu Shi

**Affiliations:** Materials Science and Engineering, College of Engineering and Applied Science, University of Cincinnati, Cincinnati, OH 45221, USA; donglu.shi@uc.edu

**Keywords:** structural complexity, Kolmogorov complexity, fractal dimension, non-equilibrium thermodynamics, system evolution, directionality, self-organized criticality, open systems, entropy beyond equilibrium, complexity science

## Abstract

We propose a universal framework for understanding system evolution based on structural complexity, offering a directional signature that applies across physical, chemical, and biological domains. Unlike entropy, which is constrained by its definition in closed, equilibrium systems, we introduce Kolmogorov Complexity (KC) and Fractal Dimension (FD) as quantifiable, scalable metrics that capture the emergence of organized complexity in open, non-equilibrium systems. We examine two major classes of systems: (1) living systems, revisiting Schrödinger’s insight that biological growth may locally reduce entropy while increasing structural order, and (2) irreversible natural processes such as oxidation, diffusion, and material aging. We formalize a Universal Law: expressed as a non-decreasing function Ω(t) = α·KC(t) + β·FD(t), which parallels the Second Law of Thermodynamics but tracks the rise in algorithmic and geometric complexity. This framework integrates principles from complexity science, providing a robust, mathematically grounded lens for describing the directional evolution of systems across scales-from crystals to cognition.

## 1. Introduction

The Second Law of Thermodynamics has long served as a foundational principle for defining the directionality of system evolution [1,2,3,4]. By asserting that entropy—A measure of disorder or energy dispersion-tends to increase in isolated systems, the law offers a time-asymmetric arrow for physical processes. From chemical reactions to cosmic expansion, entropy has traditionally been regarded as the universal metric guiding the irreversible flow of events, favoring states of higher disorder and thermodynamic equilibrium.

While entropy remains a powerful and well-established concept, its limitations become increasingly apparent when applied to real-world systems. First, entropy is rigorously defined only for idealized closed systems, whereas most natural and biological systems are open, continuously exchanging energy and matter with their environments. Second, the core notion of entropy as a “measure of disorder” is inherently vague and discipline-dependent, lacking a consistent, objective, and quantifiable definition across scientific domains. Third, due to these ambiguities and the difficulty of defining precise system boundaries, it is often unclear whether entropy has increased or decreased in any specific open system, particularly those operating far from equilibrium.

These conceptual and practical challenges highlight the need to seek alternative or complementary signatures of system evolution-ones that preserve the directional insight of the Second Law but extend its applicability beyond closed and idealized systems. In particular, there is growing interest in identifying structural indicators that can capture the emergence of organized, patterned complexity in evolving systems, especially those that maintain non-equilibrium configurations over time.

We propose such a framework in terms of structural complexity, grounded in mathematically rigorous and empirically measurable metrics: Kolmogorov Complexity (KC) [5,6,7,8,9] and Fractal Dimension (FD) [10,11,12,13,14], together referred to as the KFD model. Unlike entropy, which emphasizes disorder, these measures capture the growth of organized complexity. Kolmogorov Complexity quantifies the minimum algorithmic length needed to describe a structure, while Fractal Dimension characterizes its scale-invariant geometrical richness. This approach reveals a complementary principle of system evolution: the accumulation of algorithmic and geometrical structure in open, dynamical systems. Such directional change—toward higher KC and FD—provides a robust, quantifiable signature of system development that is not only compatible with the Second Law but also capable of describing a broader range of phenomena.

In this work, we formalize the KFD model as a general theory of structural evolution in natural systems. We argue that systems evolve not merely through the dispersal of energy and the increase in disorder, but also through the progressive buildup of structural complexity. This perspective offers a powerful alternative lens through which to understand the deep logic of change in physical, biological, and cultural systems.

## 2. Theoretical Analysis: Kolmogorov Complexity

Kolmogorov Complexity (KC) is a measure of the complexity of an object or system in terms of the shortest possible description (program) required to generate that object using a universal computational model. It is a fundamental concept in the theory of algorithmic information theory. Formally, the Kolmogorov Complexity of a string *s* is defined as the length of the shortest possible binary program *P* that can produce the string *s* on a universal Turing machine. This is mathematically expressed as [10,11,12,13,14,15,16,17]:

(1) $$K\left(s\right)=\underset{P}{\min}\left(\left|P\right|\right)$$

where K(s) is the Kolmogorov Complexity of the string *s*, P is a program (or algorithm) that produces *s*, ∣P∣ is the length of the program *P*. The Kolmogorov Complexity provides a measure of the information content of an object, with simpler systems having lower complexity (shorter programs) and more complex systems having higher complexity (longer programs).

In the context of material systems and biological entities, we can use KC to quantify their complexity. For example:

- Inorganic systems, such as crystals and minerals, can be described by simple repeating patterns or structures, resulting in low Kolmogorov Complexity.
- Organic molecules, such as proteins and DNA, exhibit higher complexity due to the intricate sequence of elements and bonds.
- Living organisms, such as plants and animals, demonstrate even greater complexity, both in terms of the information encoded in their genes and the organization of their cellular structures.

Thus, by applying Kolmogorov Complexity to systems at different scales, we can quantify the transition from simple inorganic matter to complex living organisms.

## 3. Fractal Dimension and Structural Complexity

Fractal Dimension (FD) is another metric that can be used to quantify the structural complexity of systems, particularly at scales where self-similarity and irregularity dominate [18,19,20]. The Fractal Dimension describes how the structure of an object scales with size, and it is defined as [10,11,12,13,14]:

(2) $$=\underset{\varepsilon \to 0}{\lim}\frac{\log\left(N\left(\varepsilon \right)\right)}{\log\left(1/\varepsilon \right)}\;\;\;$$

where N(ϵ) is the number of self-similar pieces of size ϵ, D is the fractal dimension, indicating the degree of complexity or irregularity of the object.

Fractal Dimension provides insights into the spatial complexity of systems. Systems with high FD values exhibit intricate, irregular structures, while those with low FD values tend to be more uniform and regular. In the context of evolution, FD can be used to measure how systems become more structurally complex over time. For instance:

- Crystalline minerals exhibit low FD due to their periodic, ordered structures.
- Trees and plants, with branching patterns and varied geometries, have higher FD values.
- Biological networks (e.g., neural or vascular systems) exhibit even higher FD due to their fractal-like nature at multiple scales.

By combining KC and FD, we can more comprehensively describe how a system’s structural complexity evolves over time, both in terms of informational content (KC) and spatial intricacy (FD).

While Fractal Dimension (FD) offers valuable insights into the spatial intricacy and scaling behavior of structures, it is important to emphasize that FD alone does not universally quantify complexity. For example, a perfectly smooth 2D surface (FD = 2) may exhibit lower meaningful complexity than a fractal curve with a smaller FD value due to differences in geometric irregularity and functional organization. Moreover, FD values depend inherently on the embedding space dimension, limiting direct comparisons across systems of differing spatial scales such as molecules, tissues, and ecosystems. Therefore, in this study, FD is employed as a context-dependent partial measure of structural complexity, to be interpreted alongside Kolmogorov Complexity (KC), which captures algorithmic and informational content. The combined KFD model integrates these complementary metrics to provide a more holistic representation of system complexity within their appropriate spatial and conceptual domains. We acknowledge the need for caution in interpreting FD and highlight opportunities for future development of scale- and dimension-independent complexity measures.

To illustrate the applicability of Kolmogorov Complexity (KC) and Fractal Dimension (FD) as quantitative metrics of structural and informational complexity, we present a range of representative systems spanning abiotic environments to advanced multicellular life. Table 1 outlines typical examples across evolutionary stages, each exhibiting increasing levels of organizational intricacy. At the lowest end of the spectrum, abiotic systems such as soil, rocks, water, and air possess highly regular or amorphous structures, reflected in their low KC and near-linear FD values (~1.0–1.2). As organic complexity emerges—from molecular aggregates in prebiotic chemistry to encoded RNA structures—both KC and FD rise, capturing the emergence of information-bearing sequences and modular assembly. With the development of cellularity and metabolism in protocells and bacteria, measurable increases in feedback regulation and spatial compartmentalization are observed. These trends continue through early plants, where photosynthesis and fractal morphology further elevate complexity, culminating in multicellular animals and ecosystems characterized by dense interconnectivity, neural processing, and nested hierarchies. This staged progression illustrates how KC and FD offer a unified and quantifiable framework for evaluating the directional growth of structural and functional complexity in evolving systems.

## 4. Application to Living Systems

In a seminal work, What is Life?, Erwin Schrödinger proposed a provocative perspective on living systems that stands in contrast to the behavior of non-living physical systems governed by the Second Law of Thermodynamics [21,22]. He observed that, unlike inanimate systems where entropy naturally increases, life maintains and even builds order by feeding “negative entropy”—a conceptual precursor to what we now understand as free energy. This ability to reduce entropy locally enables living organisms to grow, self-organize, and sustain highly ordered structures. In contrast to the mechanical universe, where entropy increase is the default trajectory, biological evolution appears to carve out a reverse path, reducing disorder as complexity increases. However, this apparent entropy reduction does not violate thermodynamic laws, as it occurs within open systems that exchange energy and matter with their environments. Importantly, Schrödinger also noted that the entropy-defying nature of life is temporary; once an organism dies and its metabolic processes cease, entropy resumes its increase as the system degrades and returns to thermodynamic equilibrium. This dynamic indicates a fundamental distinction: while non-living systems exhibit unidirectional entropy increase, living systems achieve localized entropy reduction during life, only to revert to universal thermodynamic behavior at death. This paradox highlights the limitations of entropy as a comprehensive descriptor of system evolution, especially in biologically open and dynamically structured environments. This apparent contradiction calls for an alternative framework that can reconcile biological organization with thermodynamic consistency.

Here are several more typical examples of living systems that illustrate increasing Kolmogorov Complexity (KC) and Fractal Dimension (FD) over time or across biological organization, further supporting the idea that biological complexity rises consistently even when entropy may locally decrease (Table 2). Consider a eukaryotic cell, with its multilayered organelles, encoded genetic material, and regulatory networks. The KC required to describe such a system is vastly greater than that for an inorganic crystal. During biological growth and development, entropy may locally decrease, as Schrodinger argued, due to increased structure and function. However, KC and FD consistently increase, capturing the rising complexity and information content of the system. Thus, complexity metrics offer a coherent way to describe biological evolution without conflicting with thermodynamic principles.

A single fertilized egg (zygote) divides and differentiates into a vast array of specialized cells forming tissues, organs, and systems. KC increases because the instructions and regulations required to describe the differentiated states, gene expression profiles, and intercellular communications are more complex than a single cell [23,24,25,26]. FD increases as spatial patterning and branching structures (e.g., neural networks, vascular systems) emerge during development, exhibiting higher geometrical complexity.

The human brain starts with a relatively simple architecture and develops into a massively interconnected network of billions of neurons and trillions of synapses [27,28,29,30]. KC increases as describing the full connectivity matrix, neurotransmitter dynamics, and plasticity mechanisms requires immense algorithmic information. FD increases due to the brain’s dendritic and axonal arborization, which display fractal-like spatial organization.

A plant seed develops into a mature plant with leaves, stems, flowers, and roots, each with complex internal and external architectures [31,32,33,34,35,36]. KC increases as each stage adds layers of regulatory control (e.g., phototropism, gravitropism, hormone signaling) and anatomical complexity. FD increases due to the self-similar branching patterns of root and shoot systems and leaf venation structures.

The immune system continuously adapts to environmental challenges, producing a diverse set of antibodies and memory cells through recombination and selection. KC increases with the combinatorial explosion of possible antigen receptors and memory responses encoded in the genome. FD increases in the structural organization of lymphatic tissue and dynamic cellular movement and interactions in immune responses.

The transition from unicellular to multicellular life forms represents a dramatic leap in organizational complexity. KC increases as new levels of genetic regulation, cell–cell communication, and spatial patterning are required to maintain coordinated behavior. FD increases as tissues and organs with nested, recursive, and spatially distributed functions evolve.

These examples highlight how living systems inherently move toward higher KC and FD, which capture the growth of structure, function, and information content more precisely than entropy alone. This progression offers a unifying framework for describing biological evolution and development in terms of increasing complexity-one that remains consistent with, but extends beyond, classical thermodynamic descriptions.

## 5. Application to Natural Processes

Many naturally occurring transformations proceed irreversibly and directionally, diverging from cyclic or reversible transitions like melting or solidification [35,36]. These irreversible processes often exhibit increasing structural complexity and information content [37,38,39]-trends that can be effectively captured through Kolmogorov Complexity (KC) and Fractal Dimension (FD). Natural examples include atomic diffusion, gas expansion, mineral formation, corrosion, and polymer aging. In each case, the system transitions from a simple, low-KC state to a more complex, higher-KC configuration. These changes are not just entropic in the classical sense but also informational, reflecting richer internal structure and spatial patterning (Table 3).

For example, the oxidation of iron transforms pure Fe into hematite (Fe_2_O_3_), shifting from a symmetric metallic lattice to a chemically richer, less symmetric oxide structure that requires longer algorithmic descriptions. Similarly, carbonation turns CaO into CaCO_3_, introducing complex carbonate groups and increasing both chemical and structural complexity [40,41].

Silicate weathering breaks down feldspar into clays and hydrated minerals. These products are layered, compositionally variable, and microstructurally complex-exemplifying higher FD and KC. In polymer aging, initially linear chains cross-link or fragment into heterogeneous networks, increasing topological and chemical information [42,43].

Rusting further demonstrates this principle: a smooth metallic surface evolves into a porous, multi-phase oxide layer with chemical and spatial variability. The resulting structure is richer in both KC and FD due to the formation of irregular microfeatures and compositional phases [44,45].

In atomic diffusion, atoms migrate from high to low concentration, irreversibly flattening compositional gradients. Though thermodynamically driven by entropy, this diffusion also increases KC by producing spatial distributions that require more information to fully describe. Likewise, gas expansion from high to low pressure is a textbook example of entropy increase. As molecular positions and velocities randomize, the state becomes less compressible, reflecting a rise in KC and often FD-especially when viewed through spatial pattern analysis.

Geological processes such as mineral crystallization and metamorphism follow a similar path. Over time, relatively simple chemical inputs form complex, multi-phase solids with increased structural intricacy. These transformations consume thermal and chemical gradients, leading to entropy production while simultaneously generating organized, but algorithmically rich, configurations.

These examples support a unified view: entropy, complexity, and information are not contradictory, but rather complementary. Structural complexity emerges from thermodynamic dissipation, and measures like KC and FD can track this emergence even in open or non-ideal systems-unlike classical entropy, which is often restricted to adiabatic or closed systems. The KFD model thus offers a powerful alternative: a universal, scalable framework to quantify directional evolution, applicable across physical, chemical, and biological domains. It captures not just disorder, but the emergent organization of systems as they evolve irreversibly over time.

The KFD model and the associated function Ω(t) apply primarily to systems undergoing constructive evolution, where sustained energy flux enables the accumulation of structural and informational complexity over time. Once such driving forces cease—e.g., in a closed system undergoing diffusion, or after biological death—the system enters a degenerative phase where both Kolmogorov complexity (KC) and functional differentiation (FD) can decline. This behavior is not a counterexample, but rather consistent with the model’s scope: Ω(t) is intended to characterize directional complexity growth under nonequilibrium conditions, analogous to local entropy decrease being possible only in open systems. In this context, the observed decline in Ω(t) in such passive processes lies outside the domain for which the model is formulated.

## 6. Mathematical Framework for Kolmogorov Complexity (KC) and Fractal Dimension (FD) as Functions of System Evolution over Time

Understanding the evolution of complexity in natural systems-from simple crystalline solids to highly structured multicellular organisms-requires not only qualitative descriptions but also quantitative frameworks. In this study, we apply mathematical modeling to capture the dual nature of complexity: the rise in structured complexity through biological evolution. To this end, we model the progression of two key complexity metrics-Kolmogorov Complexity (KC) and Fractal Dimension (FD)-as functions of system evolution over time.

### Structured Complexity Growth

The increase in structured complexity during evolution is modeled using a logistic growth function, a well-established mathematical form used to describe systems that exhibit saturating growth behavior. This model captures the gradual but accelerating increase in complexity during the transition from inorganic matter to life, eventually plateauing as systems reach a peak level of organization (e.g., multicellular organisms with nervous and vascular systems). The logistic function ensures bounded, smooth, and continuous growth, reflecting biological constraints and developmental saturation.

Kolmogorov Complexity (KC): [4,5,6,7,8,9]:

(3) $$KC\left(t\right)=\frac{A_{kc}}{1+e^{-k_{1}\left(t-t_{peak}\right)}}\;$$

Fractal Dimension (FD):

(4) $$FD\left(t\right)=\frac{A_{fd}}{1+e^{-k_{1}\left(t-t_{peak}\right)}}\;\;\;$$

where 

$A_{kc}=10,A_{fd}=2.5$: asymptotic maximum values during growth.

$k_{1}$ = 0.15: growth rate constant.

t_peak_ = 50: center of biological complexity emergence.

The parameters for the Kolmogorov Complexity (KC) and Fractal Dimension (FD) growth models were chosen based on conceptual consistency with observed biological and physical system development.

A*_kc_* = 10, A*_fd_* = 2.5: These values represent the asymptotic maximum complexity measures attainable during the constructive growth phase of natural system evolution. The value of 10 for KC reflects a high, yet finite, encoding complexity associated with multicellular organisms, while 2.5 for FD corresponds to the upper limit of geometrical complexity typically observed in branched and networked biological systems (e.g., vascular and neural systems).

k_1_ = 0.15: The growth rate constant was selected to reflect a moderate logistic growth dynamic, capturing the nonlinear acceleration of complexity from early inorganic stages (e.g., crystals) through biological evolution (cells to organisms).

t_peak_ = 50: This time point marks the center of the biological complexity emergence window, approximating the evolutionary transition from unicellular to multicellular life. It aligns with the inflection point of the logistic growth curve, where the rate of complexity increase is at its maximum.

Using the semi-quantitatively defined parameters of Kolmogorov Complexity (KC) and Fractal Dimension (FD), we present a biologically meaningful and mathematically tractable model that illustrates the structured accumulation of complexity in natural systems over evolutionary time. The figure charts the evolution of system complexity from crystalline solids to multicellular organisms, with each stage labeled on the *x*-axis and corresponding KC (log_10_ scale) and FD values plotted on the *y*-axis. The trajectory of complexity unfolds in two major phases: an initial physical-chemical evolution and a subsequent biological development. In the early phase, KC and FD values increase gradually. Crystalline solids such as elemental Fe and Si exhibit low complexity, reflecting their high symmetry and minimal algorithmic description. As the system progresses through inorganic compounds (e.g., Fe_2_O_3_, silicates) and simple organic molecules (e.g., hydrocarbons, polymers), complexity begins to increase modestly, driven by greater molecular diversity and structural variation [40,41,42,43,44,45]. The onset of biological systems marks a turning point. Prokaryotic cells introduce functional complexity via genetic encoding, metabolic pathways, and environmental responsiveness. Eukaryotic cells further amplify this through intracellular compartmentalization, regulatory networks, and emergent coordination [31,32,33,34]. The emergence of multicellular organisms—represented here by humans—drives a sharp rise in both KC and FD, reflecting intricate developmental programs, intercellular communication, and specialized tissue organization [27,28,29,30]. The figure thus captures a monotonic, stage-wise increase in complexity as natural systems evolve from physical order to biological function.

## 7. Toward a Universal Law of Complexity Growth

We propose that for all open, divergent systems-especially in biological and naturally evolving physical contexts-Kolmogorov Complexity and Fractal Dimension increase over time. This law is consistent with thermodynamics and offers clarity in structural evolution characterized by KC and FD. By formalizing complexity as a fundamental, quantifiable, and directional property of system evolution, we offer a complementary principle to the second law of thermodynamics-one that accounts for the real-world behavior of both living and non-living systems.

One of the central arguments in the study of natural systems-both biological and non-biological-is that evolutionary processes consistently drive an increase in structural and informational complexity. As systems evolve, their Kolmogorov Complexity (KC) tends to increase, capturing the growing intricacy of internal organization, component diversity, and interdependencies. This complexity trend offers a unifying metric for tracking irreversible transformation across physical, chemical, and biological domains.

For a general directional indicator of system evolution, we now propose the Universal Complexity Law (UCL) as:

(5) dΩ≥0

where Ω is a general complexity potential defined as a function of Kolmogorov Complexity (KC) and Fractal Dimension (FD):

(6) $$\Omega =\alpha \cdot KC\left(t\right)+\beta \cdot FD\left(t\right)$$

where *α* and *β* are scaling constants to normalize units (analogous to Boltzmann’s constant), *t* is time or a generalized progression variable (such as developmental stage). The Ω(t) complexity function is formulated as a weighted linear combination of Kolmogorov Complexity (KC) and Fractal Dimension (FD) to integrate complementary dimensions of structural and informational complexity. This linear form is selected for its conceptual simplicity and interpretability, providing a first-order approximation of overall system complexity during constructive growth phases. While alternative nonlinear or multiplicative formulations could potentially capture additional nuances, they lie beyond the scope of the present study and are promising avenues for future research. The parameters α and β are phenomenological scaling constants, introduced to balance the disparate units and magnitudes of KC and FD. Analogous to fundamental constants in physics, these parameters highlight the model’s need for empirical calibration tailored to specific systems and contexts. Importantly, Ω(t) is intended to characterize directional trends in complexity during energy-driven evolution, rather than serve as a strict invariant. Thus, localized or temporal decreases in KC or FD—reflecting pruning, degradation, or remodeling—do not contradict the model’s conceptual framework but rather define its domain of applicability.

The differential form:

(7) $$\frac{d\Omega }{dt}\geq 0$$

indicates a non-decreasing trajectory of system complexity over time in irreversible, open-system processes. This law expresses the irreversible increase in structural, informational, and geometrical complexity as observed in:

○ Evolution of living systems (life emergence, development),
○ Irreversible physical processes (oxidation, diffusion, growth),
○ Natural self-organization (fermentation, biochemical pathways).

For living systems, such as a eukaryotic cell evolve over time, its genetic code becomes more diversified (↑ KC). Its structural organization (organelles, networks) becomes more intricate (↑ FD); thus:

(8) $$\;\frac{d\Omega }{dt}>0$$

as long as it is growing or adapting.

One could add a dissipative term similar to entropy production:

(9) $$\frac{d\Omega }{dt}=\Phi \left(t\right)\geq 0$$

where Φ(t) is the complexity flux, representing the rate of complexity generation or transformation, which could be tied to energy consumption, chemical gradients, or information processing rates. Top of FormBottom of Form. This framework accommodates a wide range of transformations that exhibit increasing Ω(t), such as:

- The self-organization of lipid bilayers into protocells;
- The polymerization of monomers into functional biopolymers;
- The progression from unicellular to multicellular organisms;
- The geological formation of multi-phase rocks with interlocking grains,
- The development of dendritic structures during metallurgical solidification.

In each case, the system undergoes irreversible, information-rich transformations that leave a clear complexity signature. Thus, the proposed expression dΩ/dt ≥ 0 may serve as a universal directional law of evolution, complementing the second law of thermodynamics while remaining applicable to both living and non-living systems.

## 8. Analogy to Entropy

In classical thermodynamics, entropy quantifies the degree of disorder or the number of possible microstates a system can occupy, succinctly expressed by Boltzmann’s equation:

(10) $$S=k_{B}\ln W$$

where S is entropy, k_B_ is the Boltzmann constant, and w is the number of accessible microstates [46,47]. This formulation conveys a foundational concept: in isolated systems, entropy naturally increases, driving systems toward disorder and equilibrium. While the equation is mathematically rigorous and conceptually sound, in practice, neither S nor w can be easily measured or defined for specific systems and transformations, particularly those involving structural or chemical complexity, as illustrated in Table 3.

In an analogous way, the concept of evolutionary complexity—represented by a function Ω such as Kolmogorov Complexity (KC) or Fractal Dimension (FD)—can be used to describe the progression of systems, particularly biological or structurally evolving ones, toward higher informational and structural richness. Just as entropy increases over time in irreversible thermodynamic processes, we propose a universal directional tendency in evolving systems, expressed as d*Ω*/d*t* ≥ 0. This formulation reflects that, over time, the structural and informational complexity of systems increases, whether through biological evolution, natural selection, or physical processes such as crystal growth, diffusion, or the formation of self-organized structures. While entropy deals with the statistical distribution of states, evolutionary complexity captures the algorithmic and geometric intricacy, providing a complementary and potentially unifying measure for describing system evolution:

(11) $$\frac{d\Omega }{dt}>0\mathrm{system}\;\mathrm{evolves}\;\mathrm{to}\;\mathrm{higher}\;\mathrm{complexity}\;(e.g.,\mathrm{inorganic}\;\to \mathrm{organic}\;\to \;\mathrm{life})$$

(12) $$\frac{d\Omega }{dt}=0\to \mathrm{static}\;\mathrm{or}\;\mathrm{equilibrium}\;\mathrm{state},$$

(13) $$\frac{d\Omega }{dt}<0\to \mathrm{degradation}\mathrm{or}\mathrm{simplification}\;(e.g.,\mathrm{decay},\;\mathrm{decomposition},\;\mathrm{death}).$$

Destruction, decay, and degradation are not uniformly characterized by a loss of complexity. Instead, the behavior of complexity measures such as Kolmogorov Complexity (KC) and Fractal Dimension (FD) during such processes depends strongly on whether the system is inorganic or biological. In inorganic systems, destructive processes frequently lead to increases in KC and FD. For example, the oxidation of elemental iron produces hematite (Fe_2_O_3_), a porous, chemically heterogeneous material with irregular surface morphology. Similarly, geological weathering transforms crystalline minerals into compositionally diverse clays and oxides. These transitions result in structures that are less symmetric, more irregular, and require longer algorithmic descriptions-hallmarks of increased KC. The spatial inhomogeneity and roughness of the resulting materials also lead to higher FD values, reflecting the more complex spatial patterns formed through irreversible transformation.

In contrast, living systems often exhibit the opposite behavior during terminal stages of degradation. While biological structures are extraordinarily complex at their peak-exhibiting high degrees of functional, spatial, and informational organization-destruction through death and decomposition typically results in decreasing complexity over time. Early stages of biological decay may exhibit transient increases in KC and FD due to microbial colonization, fragmentation, and the chaotic reorganization of tissues. However, as decomposition progresses, the system converges toward uniformity: cells break down, molecules are mineralized or oxidized, and the organism ultimately reduces to simpler compounds such as water, carbon dioxide, and nitrogenous waste. These end-products are chemically and spatially homogeneous, leading to lower KC and FD values.

This distinction reveals a key difference: in inorganic materials, destruction often introduces complexity through phase separation, roughness, and heterogeneity; in living systems, destruction tends to remove organized complexity, eventually yielding a low-information, entropic state. The former results in higher descriptive and structural complexity, while the latter reflects the breakdown of function and order. Thus, increases in KC and FD are not universal markers of destruction. Rather, these complexity metrics serve as sensitive indicators of the type and trajectory of change. They capture whether a system is evolving toward a more diverse and structurally irregular state, or degrading into uniform simplicity. This distinction highlights the importance of system-specific analysis when applying complexity frameworks to natural processes, and it further underscores the complementary roles of entropy, KC, and FD in describing irreversible transformations.

## 9. Discussion

In this study, we introduced structural complexity as a quantitative and directional signature of system evolution, applicable to both inorganic and organic systems. Unlike entropy—which measures disorder and is often constrained to thermodynamic interpretations—structural complexity, captured through Kolmogorov Complexity (KC) and Fractal Dimension (FD), provides a more versatile and informative lens for examining the progressive organization and intricacy of evolving systems.

The KFD framework is intended primarily as a conceptual and semi-quantitative tool to capture the directional evolution of complexity in open, energy-driven systems. While explicit calculation of Kolmogorov Complexity (KC) and Fractal Dimension (FD) for real systems can be challenging, practical proxies exist: KC may be estimated from compression algorithms, network connectivity measures, or algorithmic reconstruction steps, and FD from box-counting, correlation dimension, or fractal analysis of spatial patterns. Importantly, Ω(t) provides a relative and continuous measure of structural and informational complexity, rather than an exact absolute value. Regarding entropy, we have updated our discussion to move beyond outdated notions of “disorder” and emphasize modern information-theoretic and non-equilibrium perspectives, highlighting that entropy and complexity are complementary: entropy quantifies the multiplicity of accessible microstates, whereas complexity captures algorithmic, structural, and spatial richness. This distinction allows KFD to provide a directional metric of evolution and organization in both living and non-living systems, bridging conceptual insights with measurable system properties.

A central argument of our work is that natural systems tend to evolve toward higher structural and informational complexity. This trend can be expressed mathematically as: dΩ/dt > 0 represents a generalized measure of system complexity, such as KC or FD. This formulation reflects a unidirectional trajectory akin to the Second Law of Thermodynamics, but is more appropriate for open, far-from-equilibrium systems where local order can increase. While entropy rises globally, complexity can increase locally—even when entropy decreases—offering a complementary and more nuanced view of system evolution.

While classical Boltzmann-Gibbs entropy has known limitations in describing the dynamics of open, complex systems, various generalizations—most notably Tsallis entropy—have been developed to address these shortcomings [48,49]. Tsallis entropy introduces a non-extensive parameter q that captures deviations from standard additivity, enabling meaningful descriptions of systems with long-range correlations, fractality, or meta-stable states. This framework has yielded profound insights into biological, ecological, and financial systems.

The proposed Ω(t) formulation does not aim to replace such entropy-based models but rather complements them by integrating algorithmic (KC) and structural (FD) complexity indicators that directly reflect patterns of organization and compressibility. Incorporating generalized entropy measures like Tsallis entropy into the Ω-framework is a promising direction for future work, potentially allowing for a hybrid formulation where α and β adapt dynamically with the system’s non-extensivity parameter Sq, thereby uniting structural, informational, and thermodynamic views of complexity. This perspective aligns with Erwin Schrödinger’s insight that living systems maintain and build internal order by exporting entropy—effectively feeding on “negative entropy” (negentropy). During biological growth and development, organisms become more structured and organized, leading to a local decrease in entropy. However, this order is achieved through energy dissipation and increased entropy in the environment, maintaining consistency with thermodynamic laws. Entropy alone, therefore, is insufficient as a universal indicator of system evolution. In contrast, complexity measures like KC and FD remain meaningful across both living and non-living systems, enabling a consistent framework for describing evolutionary pathways.

One of the most compelling extensions of this framework is the observation that mechanical softening accompanies the rise in complexity, particularly in biological systems. As matter evolves—from mineral to molecule, from cell to sentience-it becomes not only more complex but also softer and more adaptive. This softening is not a structural weakness but an evolved feature that enables flexibility, responsiveness, and functional diversity.

Biological systems-cells, membranes, and tissues-exhibit low elastic moduli (in the kilopascal to megapascal range), yet they are intricately organized and capable of self-repair, dynamic reconfiguration, and multiscale coordination. Softness supports critical biological functions such as motility, environmental sensing, and signal transduction. Hierarchical soft structures-like neurons, tendons, and plant roots-enable complex operations with minimal mechanical resistance.

Across the evolutionary spectrum, there is a clear inverse relationship between rigidity and complexity. Early Earth materials, such as rock and soil, are mechanically stiff but structurally simple. In contrast, evolved organisms exhibit lower rigidity and higher organizational complexity. This trend suggests that softness is a prerequisite for complexity and adaptability, and that evolutionary pressures favor soft, dynamic materials as a foundation for biological success.

The convergence of thermodynamic directionality, structural complexity, and material softening presents a powerful conceptual synthesis. As entropy increases, so too does the complexity of matter—particularly through the formation of soft, adaptive systems. Life, therefore, is not an isolated anomaly but a continuation of nature’s deep trajectory: a shift from static, rigid states toward dynamically complex, responsive forms.

In this work, logistic growth functions are employed as phenomenological models to represent the temporal evolution of Kolmogorov Complexity (KC) and Fractal Dimension (FD) during the constructive phases of system development. The logistic function, traditionally used to describe population growth under limiting resources, is adopted here due to its ability to capture the typical pattern of initial slow growth, followed by accelerated increase, and eventual saturation imposed by systemic constraints such as energy availability, spatial limitations, or regulatory feedback. It is important to emphasize that these logistic curves serve as conceptual tools rather than precise mechanistic descriptions. They illustrate the general boundedness and nonlinear acceleration characteristic of complexity accumulation in evolving systems, providing an analytically tractable framework for exploring directional growth trends.

We recognize that complex dynamics in natural and engineered systems often exhibit richer behaviors, including non-monotonic growth, punctuated equilibria, regression, or collapse, depending on environmental and internal factors. Our current model focuses on the idealized constructive growth phase and does not capture these more complex temporal patterns. Furthermore, the logistic parameters used herein are heuristically selected to reflect plausible timescales and saturation levels but are not derived from empirical calibration. We acknowledge the need for future empirical studies to obtain time-resolved complexity measures and for sensitivity analyses to assess parameter robustness.

This phenomenological modeling approach thus provides a useful first approximation that complements the conceptual foundation of the KFD model, highlighting the directional nature of complexity growth while leaving room for refinement and extension in future work.

Previous studies have explored step-based or generative approaches to quantify complexity, such as the “Ladder Path” method from BNU and related minimal-description frameworks, which focus on the number of construction steps or algorithmic instructions required to generate a system [50]. While these approaches capture aspects of algorithmic complexity, they generally address discrete steps or static structures rather than the continuous temporal evolution of complexity in open systems. The KFD framework extends these ideas by combining Kolmogorov Complexity (KC), which measures algorithmic information, with Fractal Dimension (FD), which captures geometrical and spatial intricacy. This dual metric allows Ω(t) to function as a directional measure of complexity growth, applicable to both living and non-living systems, and explicitly links complexity accumulation to thermodynamic fluxes and material softening. Thus, KFD provides a unified, continuous, and physically grounded perspective on system evolution that complements and extends existing generative complexity measures.

## 10. Conclusions

This study advances a unified framework for understanding the structural evolution of matter, demonstrating that the increase in entropy and the emergence of complexity are not contradictory, but rather parallel and complementary trajectories. Across diverse natural processes—oxidation, weathering, and biological development—a consistent pattern emerges: systems evolve spontaneously toward thermodynamic equilibrium (ΔG < 0), while simultaneously generating structures of increasing informational and geometrical complexity.

This duality is especially pronounced in biological systems, where a paradox unfolds: mechanical rigidity decreases even as structural intricacy increases. From the geosphere to the biosphere, this softening is not merely a biological adaptation but a broader evolutionary trajectory of matter. Hierarchical, low-rigidity structures such as tissues and cells are not exceptions to physical laws-they are their natural expressions.

By incorporating Kolmogorov Complexity (KC), Fractal Dimension (FD), and energy dissipation, we propose a generalized law of complexity growth that complements classical thermodynamics. This law formalizes the observation that systems evolve not only toward higher entropy, but also toward higher structural and informational order. It supports the philosophical proposition that life is not a statistical anomaly, but a thermodynamically driven inevitability—an emergent outcome of energy gradients and material diversity under the laws of physics.

In this light, complexity is not a rare exception but an expected consequence of the universe’s intrinsic dynamics. From galaxies to genomes, mountains to minds, the observable universe evolves not toward disorder alone, but toward structured criticality-where KC and FD increase together as hallmarks of systemic progression. The KFD Model, grounded in the principles of Self-Organized Criticality (SOC), offers a coherent theoretical framework to describe and predict this pervasive and elegant tendency.

We propose that the expressions dΩ/dt > 0 and dΩ/dt < 0 represent a universal law of structural complexity, capable of describing both the constructive and degenerative arcs of system evolution. This framework integrates thermodynamic, informational, and geometrical perspectives, offering a powerful tool for interpreting the trajectories of both living and non-living systems across physical and abstract domains.

## Figures and Tables

**Table 1:** Heuristic Complexity Estimates at Various Evolutionary Stages.

System Stage	Description	KC (Heuristic)	FD (Heuristic)	Ω(t) (Heuristic Trend)
Inert Gas	Atomically simple, no internal structure	Low (~1–2)	~1.0	Minimal
Simple Molecule	e.g., H_2_O or CO_2_	Slightly higher	~1.1	↑ Slightly
Organic Compound	e.g., glucose	Moderate	~1.3	↑↑
Functional Protein	Folded, active biomolecule	High (~10–20)	~1.6	↑↑↑
Living Cell	Self-replicating, metabolically active	Very high	~1.8	↑↑↑↑
Multicellular Organism	Differentiated systems	Higher still	~1.9	Peak Ω (constructive)

Note: Arrows in the Ω(t) column indicate relative increases in organizational complexity, with more arrows signifying stronger upward trends.

**Table 2:** Progressive increases in Kolmogorov Complexity (KC) and Fractal Dimension (FD) across various biological systems and developmental processes.

Living System	Description	KC Increase	FD Increase
Zygote	Differentiation into tissues, organs, and systems	More regulatory networks	Higher spatial complexity
Brain	Neuronal growth and synapse formation	Huge increase in algorithmic information	Dendritic and axonal arborization exhibit fractal-like
Plant	Seed developing into a mature plant	Morphogenesis, tropisms, and hormonal signaling increase	Fractal patterns in root/shoot branching and leaf venation
Immune System	Antibody diversity and memory cell formation	Combinatorial expansion from gene recombination	Structural complexity in lymphatic tissues, dynamic immune interactions
Evolution to Multicellularity	Transition from unicellular to multicellular life	New genetic, signaling, and organizational levels	Tissues/organs form with recursive and hierarchical structures

**Table 3:** Examples of natural and material processes that lead to increased structural complexity.

Process	Initial State	Final State	Change in KC
Oxidation of Fe	Pure metal (Fe)	Hematite (Fe_2_O_3_)	↑ Increased
Carbonation	CaO	CaCO_3_ (limestone)	↑ Increased
Silicate weathering	Feldspar	Clays, hydrated minerals	↑ Increased
Polymer aging	Linear chains	Cross-linked or fragmented chains	↑ Increased
Rusting	Homogeneous surface	Multi-phase oxides, porosity	↑ Increased

Note: The upward arrow (↑) in the “Change in KC” column indicates an increase in Kolmogorov Complexity.

## Data Availability

All data supporting the findings of this study are contained within the article.
